# Deep learning-based technique for investigating the behavior of MEMS systems with multiwalled carbon nanotubes and electrically actuated microbeams

**DOI:** 10.1016/j.mex.2025.103337

**Published:** 2025-04-28

**Authors:** Muhammad Amir, Jamshaid Ul Rahman, Ali Hasan Ali, Ali Raza, Zaid Ameen Abduljabbar, Husam A. Neamah

**Affiliations:** a Abdus Salam School of Mathematical Sciences, Government College University, Lahore 54600, Pakistan; b Department of Mathematics, College of Education for Pure Sciences, University of Basrah, Basrah, 61004, Iraq; c Institute of the Mathematics, University of Debrecen, Pf. 400, H-4002, Debrecen, Hungary; d Technical Engineering College, Al-Ayen University, Thi-Qar 64001, Iraq; e Department of Business Management, Al-Imam University College, Balad 34011, Iraq; f Department of Mathematics, Minhaj University Lahore, Pakistan; g Department of Computer Science, College of Education for Pure Sciences, University of Basrah, Basrah 61004, Iraq; h Department of Electrical Engineering and Mechatronics, Faculty of Engineering, University of Debrecen, 4028, Debrecen, Hungary

**Keywords:** Neural network, Optimizers, Microelectromechanical systems, Multiwalled carbon nanotubes, Electrically actuated microbeam, Deep Neural network

## Abstract

This paper proposes a model of a doubly clamped electrically actuated microbeam, a structure frequently utilized in microelectromechanical systems (MEMS). The model investigates the effect of electrostatic forces on the deflection of the beam, based on the Euler-Bernoulli beam theory. The Galerkin technique is employed to calculate the beam's deflection, while the parallel plate capacitor model simulates the electric field. We also evaluate the performance of multi-walled carbon nanotubes (MWCNTs) in MEMS. MWCNTs are promising for MEMS applications due to their significant thermal, mechanical, and electrical properties. However, predicting the behavior of these systems is challenging due to their stiffness, parametric sensitivity, and non-linearity. Deep learning strategies for handling dynamical systems are a rapidly emerging field of research. In this paper, we employ a machine learning method, called deep neural networks (DNN), to solve the non-linear systems that arise in MEMS. The primary aim of this study is to investigate the nonlinear vibration properties of MEMS oscillators, specifically those related to nanotubes and electrically actuated microbeams, using DNN algorithms. Different optimizers are used to analyze the performance and capability of these non-linear dynamical models. Numerical simulations and graphical demonstrations are presented to verify the accuracy and efficiency of the algorithm.

• The study develops a novel DNN-based model to solve non-linear systems in MEMS, particularly for oscillators with MWCNTs.
• Deep learning optimizers are applied to improve the accuracy and efficiency of predicting MEMS behavior.
• Numerical simulations confirm the effectiveness of the proposed methodology.

## Graphical abstract

**Figure:**
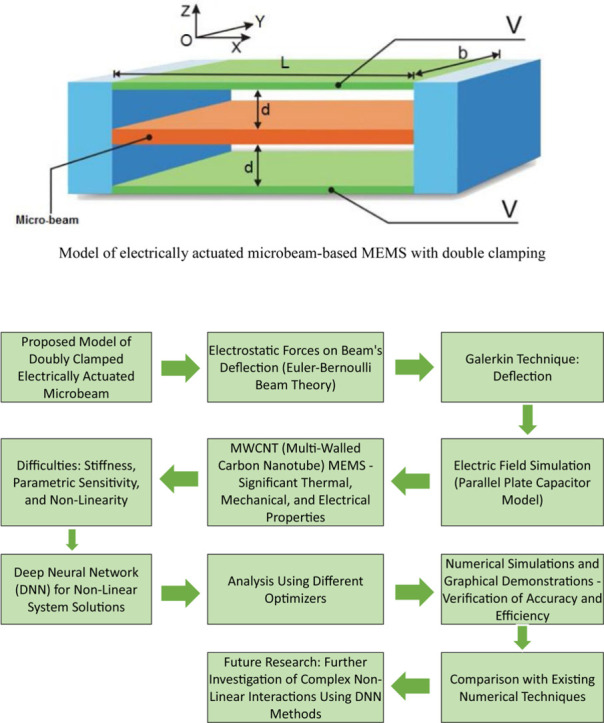

Specifications table

**Table:** 

Subject area:	Mathematics and Statistics
More specific subject area:	Dynamics of the nano/microelectromechanical systems, Deep Learning technique, Neural network, Optimization
Name of your method:	Deep Neural network
Name and reference of original method:	Artificial neural networks, ref: Lagaris, I. E., Likas, A., & Fotiadis, D. I. (1998). Artificial neural networks for solving ordinary and partial differential equations. *IEEE transactions on neural networks, 9*(5), 987–1000.
Resource availability:	Rahman, J. U., Danish, S., & Lu, D. (2024). Oscillator Simulation with Deep Neural Networks. *Mathematics, 12*(7), 959.

## Background

Nonlinear vibrations can be identified in a variety of scenarios, including molecular vibrations and earthquakes. These vibration properties are crucial in nanotechnology [[1], [2], [3]], influencing processes that involve oscillatory attachment in the controlled fabrication of nanofiber membranes [4,5], Fangzhu's oscillations to obtain water from the air [6], discharge oscillations for ion delivery [7], and oscillations in capillaries for moisture transfer [8]. Due to the rapid development of nanotechnology, the vibration challenges associated with nanotubes in nanostructures and systems have recently received a lot of attention [[9], [10], [11]]. In recent years, carbon-based nanostructures, such as graphene and carbon nanotubes, have gained significant scientific interest due to the novel mechanical, optical, chemical, and electrical properties. Moreover, these carbon-based materials are ideal for sensing applications in Nano- and microelectromechanical (MEMS) systems due to their high surface-to-volume ratios. The efficiency and design of nanotube-based MEMS and resonators are highly influenced by their conditions for periodic solutions and the properties of these solutions.

Dynamic pull-in instability is a significant phenomenon related to nano/microelectromechanical (N/MEMS) devices and is depend by certain parameters. For the useful operation and validity of such devices, analyzing pull-in instability is crucial. Investigating the vibratory dynamics of N/MEMS is challenging due to their zero total energy of these systems. Another major challenge for N/MEMS scientist and engineers is the occurrence of strong non-linearities in these systems. Nonlinearities are mostly caused by forces in actuation and detecting devices. N/MEMS are subjected to several forces such as van der Waals attraction, electrostatic force, Casimir force and electromagnetic force. In these scenarios, obtaining a periodic solution is difficult task. The most critical phenomenon in electrostatically actuated MEMS is pull-in instability. This occurs when the applied voltage surpasses a crucial value, known as the pull-in voltage. This phenomenon, first investigated by Nathanson et al. [12] and Taylor [13], is desirable in certain devices like RF switches but limits the stability range in some microbeams [14]. In a study on pull-in instability in MEM resonators, Nayfeh et al. found that the system behaved differently with AC and DC loads. Hung et al. analyzed two elements that can impact the stability region in order to expand it [15]. Bistability is another valuable feature in MEMS, enabling applications like, micro-mechanical memory and micro-based switches. When the load on a bistable framework surpasses a critical value, its geometry moves to another stable position. Qiu et al. [16] investigated this phenomenon utilizing double-clamped beams. Over the past decade, numerous studies have been conducted on snap-through buckling and pull-in instability in MEMS [[17], [18], [19]]. Krylov et al. investigated an initially curved microbeam both theoretically and experimentally, employing reduced-order Galerkin and lumped modeling of shallow arches [20]. Meguid and Chen [21] investigated snap-based buckling and pull-in instability in electrostatically microbeams, with the effect of intermolecular forces. Naveed Anjum et al. studied the dynamic pull-instability in M/NEMS by using well-known analytical technique [22].

Microelectromechanical systems (MEMS) demonstrate an extensive area of research with significant potential for the utilization of micro instruments [23,24]. MEMS have become more important to scientists and industry in the development of microstructures because of their unique and excellent properties, which include small sizes, low power consumption, a consistent appearance, and batch fabrication abilities [25]. Pull-in instability and periodic behavior are two significant phenomena in MEMS dynamics, and differential equations are a suitable tool for modeling these nonlinear properties. MEMS models are inherently nonlinear, solving them analytically is sometimes a difficult task. There are several nonlinear equations for which there are no analytical solutions [26,27]. Over the past few decades, a large number of scientists have been constructing various analytical approaches to tackle the nonlinear oscillation of MEMS. Moreover, a variety of analytical methods are applied during research to determine the solution for nonlinear models, for example, the variational iteration method (VIM) [28], residual harmonic balance technique [29], frequency formulation tool [30] Iteration Perturbation technique [31]. The traditional methods of oscillatory theory have many drawbacks when applied to the case of these microstructures. Since existing techniques are insufficient for solving highly non-linear models, several new approaches have been introduced to overcome such challenges. Due to the difficulties of accurately finding their solutions and the limited capacity of numerical techniques to clearly illustrate the frequency-amplitude relation.

Kacem et al. [32] have studied the large-amplitude non-linear vibrations of N/MEMS resonant sensors near their basic resonance. They developed a Multiphysics model utilizing the Galerkin decomposition technique and the averaging approach for electrostatically operated clamped-clamped resonators. Vyasarayani et al. [33] established a mathematical framework for an electrostatic MEMS beam that impacts with a stationary electrode after pull-in. Abedinna-sab et al. [34] studied the effect of pre-tension and compression in Euler–Bernoulli microbeams. Y.Yan et al. [35]. analyzed the dynamic behavior of multi-walled carbon nanotube (MWCNTs) using N-mode Galerkin approach. Kyungjae Yun et al. [36] explored the stability and vibrational analysis in MWCNTs by employing extended Galerkin technique. Kun Huang et al. studied the Euler Bernoulli beam theory to model and investigate the CNTs [37]. Wei Tang et al. predict the behavior of N/MEMS using variational iteration approach [38]. The governing model in this paper is determined by Euler Bernoulli beam and then applying Galerkin technique to convert this equation into ordinary differential equation (ODEs). The stability and dynamical analysis of double walled carbon nanotubes (DWCNTs) are studied by Vassil M. Vassilev et al. [39].

Therefore, to overcome such limitations, researchers have also been developing ANN models to approximate the solutions of dynamical problems. In recent years, a variety of techniques are employed to model, simulate, solve, and analyze the stability of dynamical systems, neural network-based approaches to approximate the solution of differential equations that occur in different structures have attracted interest in recent years. Deep neural networks have been effectively applied to solving nonlinear initial or boundary value problems. The classical neural network is made up of different perceptron combinations the structure known as a multilayer perceptron (MLP). The main purpose of the MLP neural network is to build a model that can handle complicated problems from huge amounts of data and with numerous variables that are incomprehensible to humans. Different ordinary differential equation (ODE) and partial differential equations (PDE) [40,41] algorithms have been established to predict neural solutions of boundary and initial value problems. The artificial neural network technique (ANN) is used to solve boundary value problems with arbitrary boundary conditions [42]. The radial basis function neural network [43] approach finds the solution to a differential equation. The approximate results of Emden fowler type equation are obtained by the Chebyshev neural network method [44]. Symplectic artificial neural network [45] analyzes the duffing oscillator model. A comprehensive analysis of the Selkov model [46] is demonstrated by a deep neural network (DNN). Motivated by all of the above discussion, it is natural to propose a DNN algorithm to solve the MEMS model of a doubly clamped electrically actuated microbeam and multi-walled carbon nanotube (MWCNT).

## Method details

### Neural methodology for differential equations

An autonomous system with nth-order ordinary differential equations (ODEs) can be expressed as

(1) $$\eta \left(\xi ,u\left(\xi \right),u^{\prime }\left(\xi \right),u^{\prime \prime }\left(\xi \right)\ldots .\;u^{\left(n\right)}\left(\xi \right)\right)=0,\;:\;\xi =\left(\xi_{1},\xi_{1},\ldots .\xi_{n}\right)\in \Psi \subset \;R^{n}$$

Where u(ξ) is expressed as the computed solution and Ψ denotes the discretized domain respectively. Suppose $u_{1}\left(\xi ,\delta \right)$ represents the artificial neural network (ANN) approximate solution to adjustable parameters such as, weights and biases. Then we can rewrite Eq. (1) as

(2) $$\eta \left(\xi_{i},u_{1}\left(\xi_{i},\delta \right),u_{1}^{\prime }\left(\xi_{i},\delta \right),u_{1}^{\prime \prime }\left(\xi_{i},\delta \right)\ldots .\;u_{1}^{\left(n\right)}\left(\xi_{i},\delta \right)\right)=0.$$

Eq. (2) can be transformed into an unconstrained optimization problem, which is significantly easier to tackle to yield the corresponding cost function of the ANN. It can be written as follows

(3) $$E\left(\delta \right)=Min\sum_{\xi_{i}\in \Psi }^{n}{\left(\eta \left(\xi_{i},u_{1}\left(\xi_{i},\delta \right),u_{1}^{\prime }\left(\xi_{i},\delta \right),u_{1}^{\prime \prime }\left(\xi_{i},\delta \right)\ldots .\;u_{1}^{\left(n\right)}\left(\xi_{i},\delta \right)\right)\right)}^{2}.$$

The approximate solution of the ANN can be represented as the sum of two terms.

(4) $$u_{1}\left(\xi_{i},\delta \right)=A+F\left(\xi ,NN\left(\xi ,\delta \right)\right),$$

Where A is the approximate solution related to the initial or boundary condition, instead of the adjustable parameters. The approximate solution of the second component NN(ξ,δ) is the output of a feed-forward neural network (FFNN), which includes M hidden layers with k neurons in each hidden layer and an input $\xi \;\in R^{n}$. This term involves the use of a neural network, the weights and biases of which must be modified to tackle the minimization problem. Observe that the problem has now been simplified from the original constrained optimization problem to an unconstrained one, making it significantly simpler to solve. The outcomes of FFNN can be expressed as follows:

(5) $$NN\left(\xi ,\delta \right)=\sum_{j=1}^{k}u_{j}\;\frac{1}{1+\text{exp}\left(-h_{j}\right)}$$

And,

(6) $$h_{j}=\sum_{i=1}^{n}w_{ji}\xi_{i}+b_{j},$$

where k is the number of neurons and $w_{ji}$ and $u_{j}\;$are the weights from the input unit i to the hidden unit j and the hidden unit j to the output unit, respectively.

#### Structural configuration

NeuroDiffEq, a Python library built on PyTorch, with the use of artificial neural networks (ANNs) to handle a wide range of differential equations. NeuroDiffEq [47] simplifies problem-solving by emphasizing defining the problem domain to establish initial or boundary conditions and differential equations. It gives users the flexibility to investigate different solution approaches, such as various ANN structures and training parameters. Instead of being restricted to fixed architecture, NeuroDiffEq is made to be flexible, enabling users to build different kinds of neural networks. In our method, we employ a Fully Connected Neural Network (FCNN), which is a fundamental form of Deep Neural Network (DNN), to approximate dependent variables. The FCNN is organized such that every neuron in a layer is connected to all neurons in the next layer, ensuring uninterrupted information flow and creating a direct and densely interconnected structure.

**Fig. 1:**
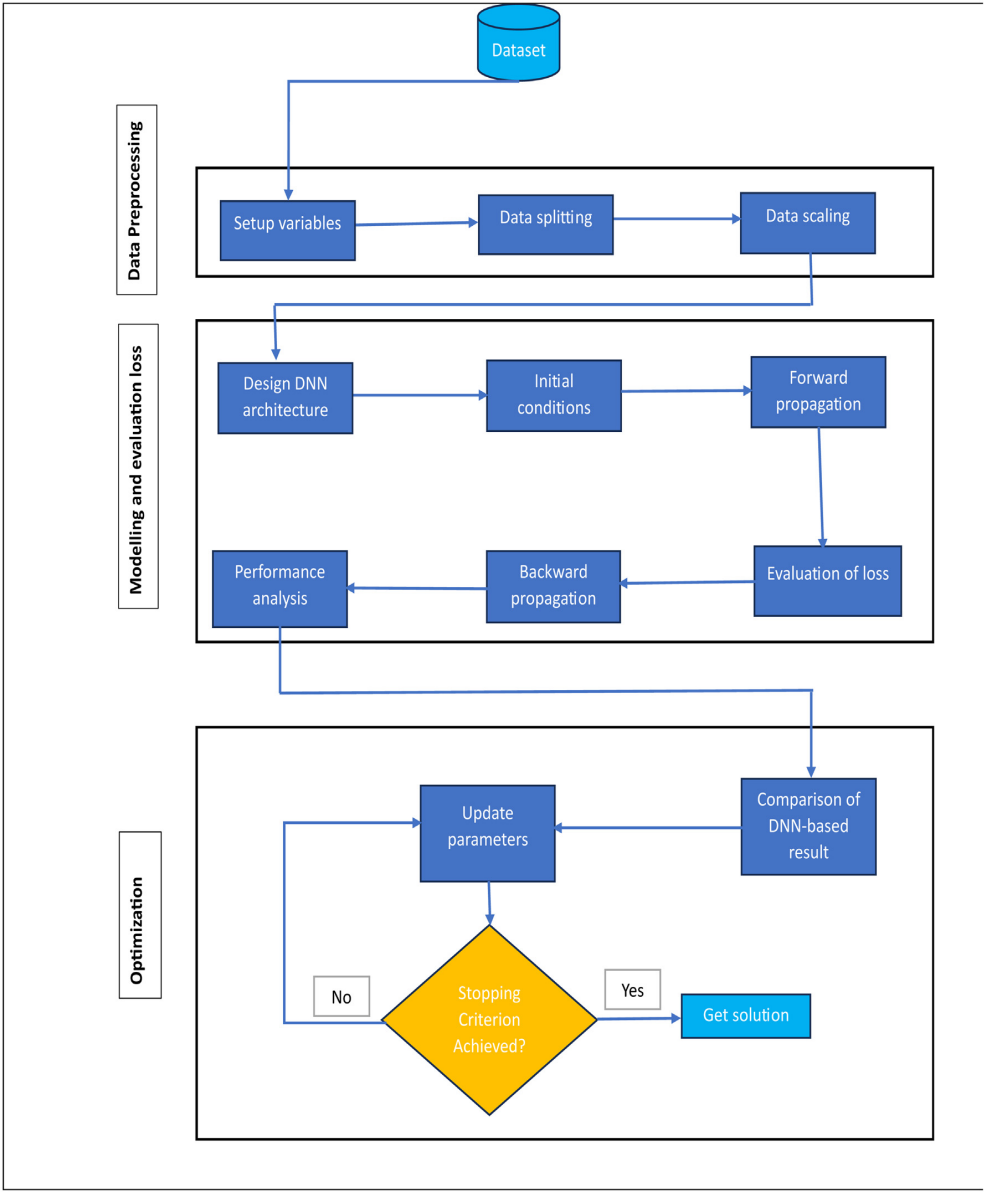
A framework of the DNN model for solving microelectromechanical systems (MEMS).

This network architecture enables thorough information propagation throughout the network, as it allows unrestricted connections between neurons across different layers. Using NeuroDiffEq to evaluate the mathematical models of Euler Bernoulli beam discussed above, we conducted several experiments. According to the basic principle of NeuroDiffEq, after the network receives the input values, the NN are updated to identify the underlying patterns in the data produced by the differential equations by employing a trial solution that also handles the initial and boundary conditions. The differential equation is expressed like an optimized problem [48] that needs to be minimized. To obtain the predicted solution of the differential equation, the trial solution is input into the differential equation's residual, which is then reduced as much as possible (Fig. 1).

This network architecture enables thorough information propagation throughout the network, as it allows unrestricted connections between neurons across different layers. Using NeuroDiffEq to evaluate the mathematical models of Euler Bernoulli beam discussed above, we conducted several experiments. According to the basic principle of NeuroDiffEq, after the network receives the input values, the NN are updated to identify the underlying patterns in the data produced by the differential equations by employing a trial solution that also handles the initial and boundary conditions. The differential equation is expressed like an optimized problem [48] that needs to be minimized. To obtain the predicted solution of the differential equation, the trial solution is input into the differential equation's residual, which is then reduced as much as possible (Algorithm 1).

**Algorithm 1:** The FCNNs algorithm for finding the solution of MEMS model.

**Preparation of data** # Import and divide the dataset (Y, Z) into sets for testing and training. $Y\_\text{train},Y\_\text{train},Z\_\text{train},Z\_\text{train}=\text{train}\_\text{test}\_\text{divide}(Y,Z,\text{test}\_\text{size}=m_{1},\text{random}\_\text{state}=m_{2}$ **Implementing the model** # Configuring the parameters of the FCNN. $\text{input}\_\text{units}=Y\_\text{train}.\text{shape},M_{1}$ $\text{output}\_\text{units}=M_{2}$ $\text{hidden}\_\text{units}=[M_{3},M_{4},M_{5}]$ Learningrate=L Epochs=E Visual_frequency=F $W=\text{initialize}\_W(M_{1},\left[M_{3},M_{4},M_{5}\right],M_{2})$ $B=\text{initial}\_B(\left[M_{3},M_{4},M_{5}\right],M_{2})$ **Forward Propagation** According to epochs in range: model_output,hidden_layer_output=Forwardpropagation(Y_train,W,B) U=A(modeloutput,A_function) **Computing the Loss** L=compute_L(z_train,U,L_func **Backward propagation** G=compute_G(Y_train,hidden_layer_output,Z_train,U) **Optimization** W,B=adjust_parameters(W,B,G,L) # A visual representation ifE%F==0 $\text{print}(f\prime \text{epoch}:\left\{E\right\},L\;{\left\{L\right\}}^{\prime })$ **Evaluation of the model** Hidden_layer_output,test_prediction=forwardpropagation(Y_test,W,B) TestL=compute_L(Z_test,testpredictions,L_function) accuracy=estimate_accuracy(Z_test,test_estimation) $\text{print}(f^{\prime }\;\text{average}\;\text{test},{\left\{\text{tes}t_{.L}\right\}}^{{}^{\prime }}).$ $\text{print}(f^{\prime }\;\text{accuracy},\;\left\{\text{Accuracy}\right\}{\%}^{{}^{\prime }}).$ **Prediction** #Give input information for prediction unknown_data=import_unknown_data U=estimate_output(unknown_data,W,B) $\text{print}(U\;\text{for}\;\text{known}\;\text{data}:{\left\{U\right\}}^{{}^{\prime }})$ Activation function = A, Estimated output= U Weight= W Gradient= G Biases= B

The structural setting of the network refers to several parameters such as the number of layers, units inside each layer, epochs, optimizer, activation function [49], loss function, and learning rate. The process involves the following steps:

I. **Prepare Data** In order to train the fully connected neural network (FCNN) using a variety of input signals and operating conditions, provide a dataset (X, Y) that comprises input X and associated dependent variable Y. Separate the dataset into training and testing folders to ensure the model performs accurately. This dataset contains problems that range widely in terms of amplitudes, frequencies, and nonlinearities.
II. **Implementing the model** Set the FCNN model parameters, such as weights, number of epochs, biases, and learning rate. the number of input units according to the input characteristics and select the number of output units in the layer based on the dependent variable. Our proposed DNN model has a single input unit because each DE demonstrated in section 3 has only one independent variable. Our network is a deep neural network because it involves three hidden layers. Each hidden layer contains a total of 64 neurons in case 1 and 32 neuron units in case 2.
III. **Forward propagation** Forward propagation must be used for each epoch to achieve the output of the given model, using activation function applied to introduce nonlinearity. The selection of the Sine activation function provides smooth and continuous training during the process. The smoothness of the sine function enables optimization efficiency and helps prevent issues like disappearing gradients, which can stop training. In addition, it improves the rate of convergence.
IV. **Computing the Loss** Utilizing a suitable loss function [50], to determine the loss that exists between the true and estimated values of the training data set. The L2 [51] loss function is utilized to compute the residuals, which is the average of the square difference among the true and estimated values.
V. **Backpropagation** we compute the gradients of the weights and biases in terms of loss, by using backpropagation [52] to propagate the gradients backward and update the weights and biases consequently.
VI. **Optimization** Update the parameters using an optimization [53] technique. The FCNN is trained with the dataset utilizing state-of-the-art techniques, such as regularization strategies, to enhance performance and generalization abilities. This step facilitates reducing the loss that was computed throughout the training procedure. The training process utilizes Adam algorithm with a learning rate set at 0.001. It operates on unique points generated for each epoch by introducing Gaussian noise [54] to evenly spaced points across the domain of t.
VII. **Evaluation of the model** Once the model has been trained for a chosen number of epochs, use a testing set to evaluate its performance. This analysis should include computing the average loss and accuracy of the proposed model.
VIII. **Prediction** After the training process is completed, pass the input characteristics to predict the output of the dependent variable for data that has not been seen before.

### Method validation

In this section, we discussed the simulation outcomes obtained with the proposed DNN technique. We performed many experiments to see how the solution of a system of differential equations can be affected by modifications of the MEMS model. These experiments are performed in Python 3.0 using the Jupyter Notebook interface.

In this section, we consider a non-linear differential equation, which illustrates various microelectromechanical systems (MEMS) utilized in nanotechnology.

(7) $$\left(g_{0}+g_{1}u+g_{2}u^{2}+g_{3}u^{3}+g_{4}u^{4}\right)u^{\prime \prime }+g_{5}+g_{6}u+g_{7}u^{2}+g_{8}u^{3}+g_{9}u^{4}+g_{10}u^{5}+g_{11}u^{6}+g_{12}u^{7}=0$$

(8) $$u\left(0\right)=A,\;u^{\prime }\left(0\right)=0$$

Where the coefficients $g_{j}\left(j=0,1,2,3,\ldots 12\right)$ are determined by applying the Galerkin method to transform the partial differential equation into an ordinary differential equation and A represents the initial amplitude of the nonlinear oscillatory system.

Case 1 Electrically actuated microelectromechanical systems (MEMS)

The simulation section concludes with an investigation of the MEMS model with different electric excitation, and the equation can be formulated as,

(9) $$\left(a_{0}+a_{1}u^{2}+a_{2}u^{4}\right)u^{\prime \prime }+a_{3}u+a_{4}u^{3}+a_{5}u^{5}+a_{6}u^{7}=0$$

(10) $$u\left(0\right)=A,\;u^{\prime }\left(0\right)=0$$

**Fig. 2:**
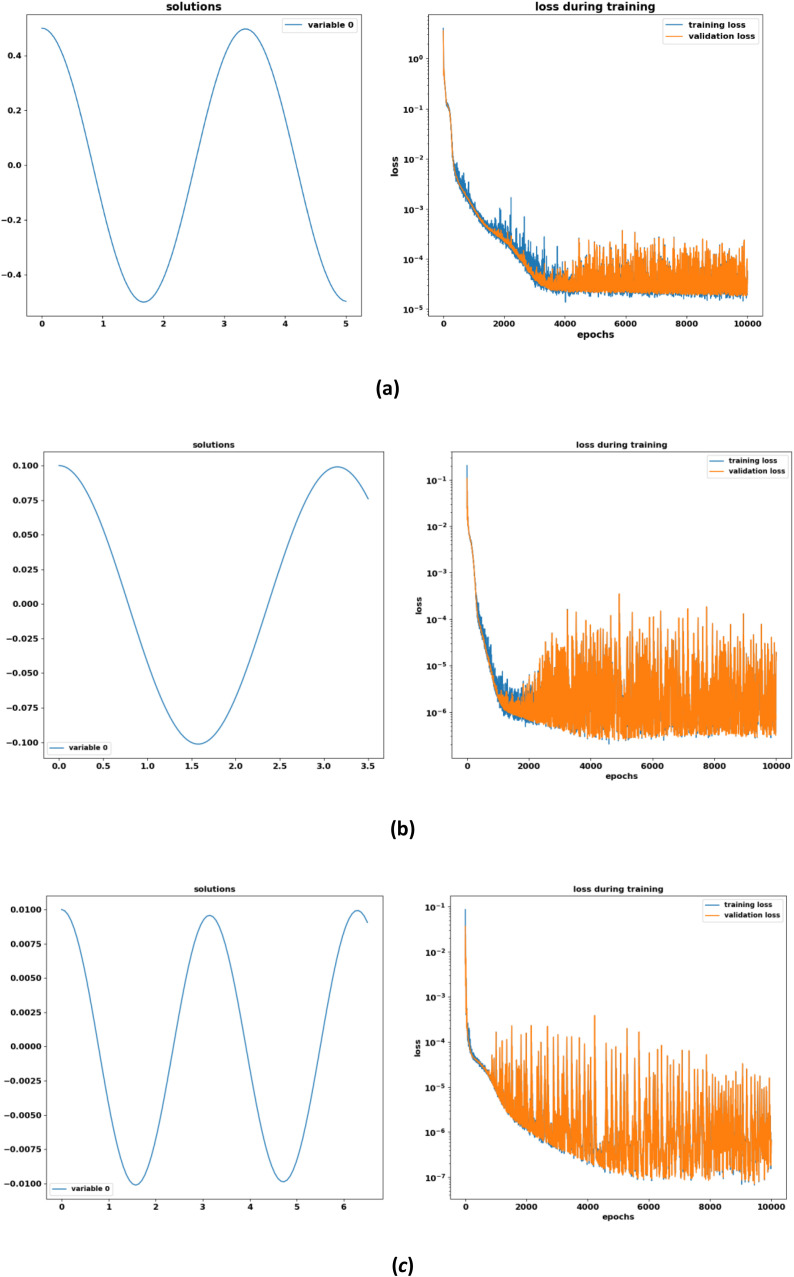
DNN-based approximated solution of Eq. (9) and training validation loss for **(a)***A* = 0.5 **(b)***A* = 0.1 **(c)***A* = 0.01.

The coefficients $a_{j}\left(j=0,1,2,3,\ldots 7\right)$ are given in Appendix A. By adjusting the parameters $g_{1}=g_{3}=g_{7}=g_{9}=g_{11}=0,\;g_{0}=a_{0},\;g_{2}=a_{1},g_{4}=a_{2},g_{6}=a_{3},g_{8}=b_{4},\;g_{10}=a_{5},\;and\;g_{12}=b_{6},$ we can obtain this oscillatory equation from Eq.(7). In Fig. 2, the left column indicates the results obtained by the DNN technique and the right column shows the training and validation loss of the suggested problem. First, we trained the network for 10,000 epochs in the case of the Adam optimizer, and graphical results are displayed in Fig. 2. It may be observed that the loss function tends to decrease with the increase of the training period.

The solution of MEMS systems using deep learning algorithms provides an efficient and accurate technique for predicting system behavior, overcoming the limits of traditional analytical and numerical methods. Complex nonlinear dynamics of MEMS devices can be accurately analyzed through neural networks and machine learning techniques with reducing computational costs. To ensure the precision and reliability of the designed sensors, it is critical to assess the variations in theoretical, numerical, and experimental data during the sensor design and fabrication process. These deviations could be caused by fabrication tolerances, material property variations, residual stresses during manufacturing, environmental impacts, or assumptions made in theoretical and numerical modeling. A comprehensive analysis of these results reveals potential differences and provides ideas into improving sensor fabrication procedures, material choices, and modeling accuracy. Moreover, integrating deep learning into sensor design can improve performance by enabling for data-driven validation, compensation for fabrication-induced errors, and adaptive optimization of sensor responses. Addressing these variations through a systematic evaluation ensures that the proposed model is both robust and practically applicable for real-world MEMS sensor applications, resulting in more reliable and efficient sensor designs.

**Fig. 3:**
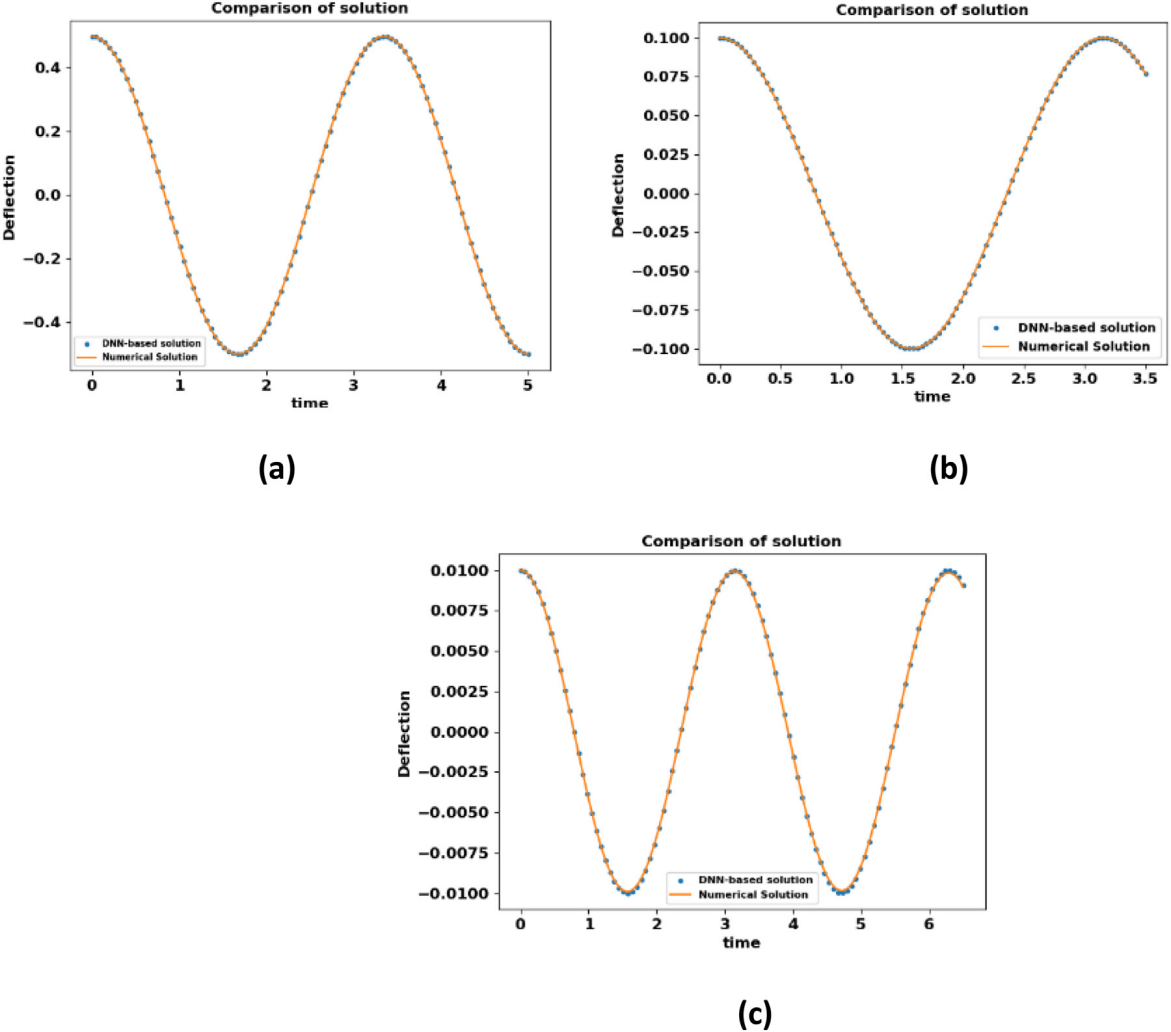
Results comparison with the DNN-based approach and numerical technique of electrically actuated microbeam.

A comparison of the solution of the electrically actuated MEMS model determined by the DNN method and numerical technique (RK4) is plotted in Fig. 3. In Fig. 3 the left column indicates the solution of neural network approximation (dotted line) and numerical method (RK4) approximation (solid lines) and this comparison demonstrates that there is an excellent agreement between the computational findings from RK4 and the approximate results obtained by the DNN approach. These figure not only indicates the consistency of the results obtained but are also useful in designing the electrically actuated MEMS model.

Case 2 Multi-walled carbon nanotubes based microelectromechanical systems (MEMS)

Consider the equation of motion for a nonlinear vibratory system.

(11) $$u^{\prime \prime }+b_{0}+b_{1}u+b_{2}u^{2}+b_{3}u^{3}+b_{4}u^{4}=0$$

(12) $$u\left(0\right)=A,\;u^{\prime }\left(0\right)=0$$

Where the parameters $b_{n}\left(n=0,1\ldots ..4\right)$ for this suggested model can be determined in Sedighi and Daneshmand [55]. By Choosing $g_{0}=1,\;g_{1}=g_{2}=g_{3}=g_{4}=g_{10}=g_{11}=g_{12}=0,\;g_{5}=b_{0},g_{6}=b_{1},g_{7}=b_{2},g_{8}=b_{3},\;and\;g_{9}=b_{4}$, we can obtain this vibratory equation from the generalized Eq.(7).

**Fig. 4:**
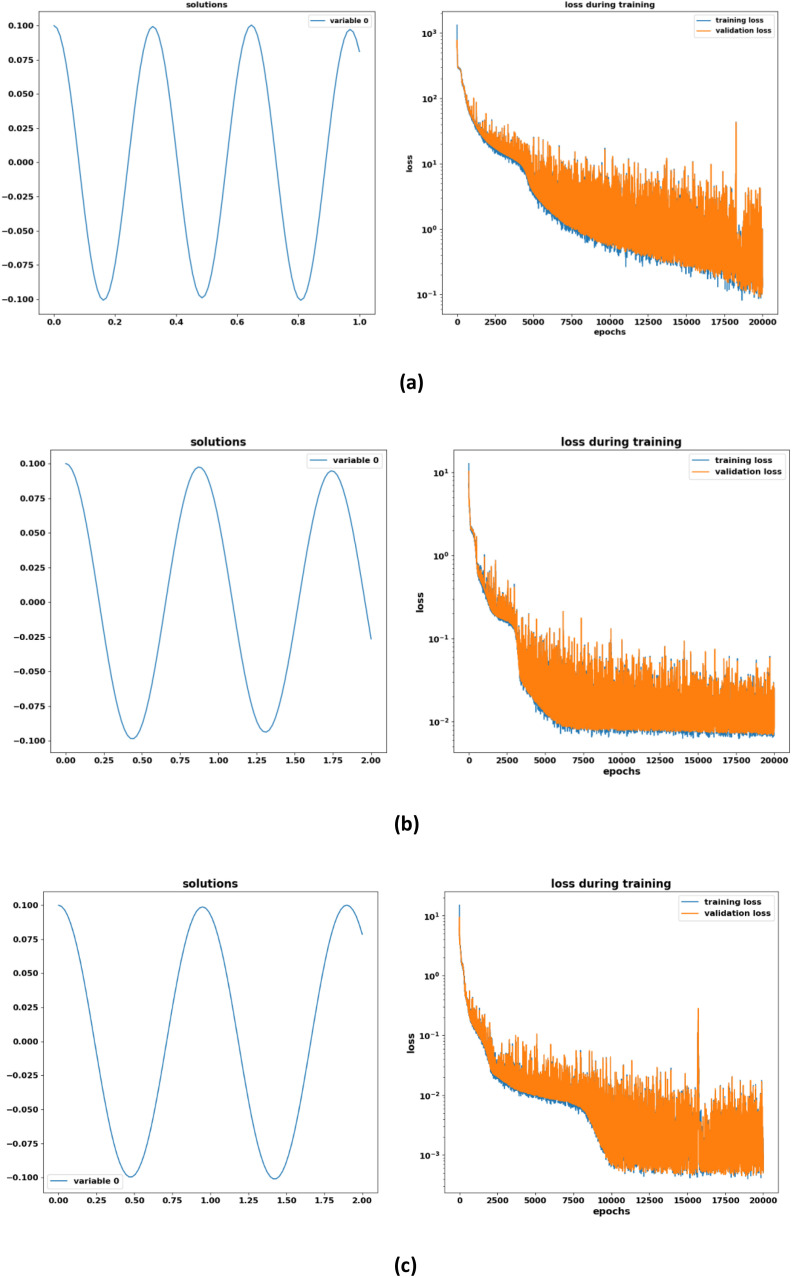
DNN based solution of multi walled carbon nanotubes MEMS model in case of different time domain and initial conditions **(a)***A* = 0.5**(b)***A* = 0.1 **(c)***A* = 0.01**.**

In Fig 4 the left column shows the predicted solution multi walled carbon nanotubes of the MEMS model obtained by the suggested DNN method and the right column indicates the training and validation loss against 20,000 epochs. The graph of training and validation loss over epochs shows the accuracy rate of DNN. It may be observed that the loss function tends to decrease with the increase of the training period.

**Fig. 5:**
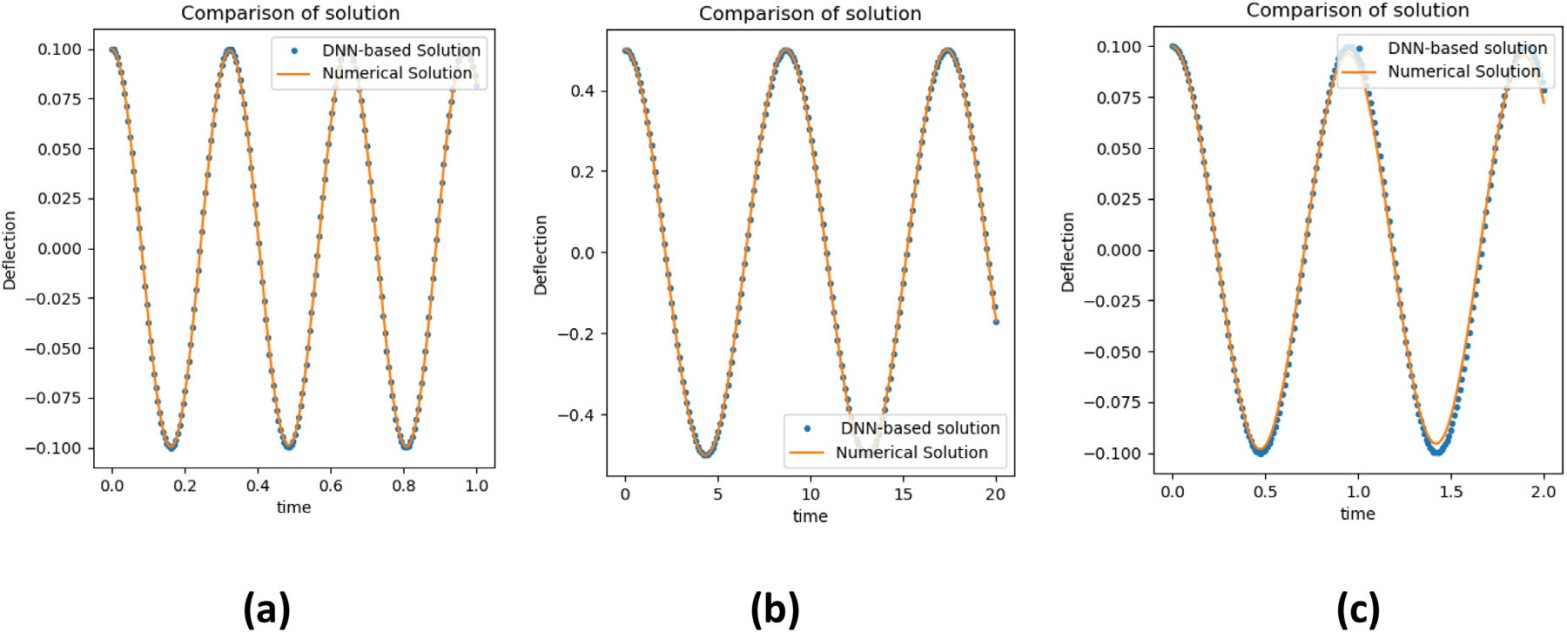
Comparison between DNN based algorithm and RK4 method for MWCNTs of MEMS model.

Fig. 5 plots the variation of multi walled carbon nanotubes for the MEMS model. Amplitude obtained from DNN results using Python (solid line), and numerical approximation determined by RK4(dotted line) is displayed in the left column of Fig 5 that confirms the accuracy of the purpose technique. Runge–Kutta and other numerical techniques are computationally expensive to use particularly for complex systems or large-scale problems. Furthermore, when more iterations are required to find a solution, the computational cost increases. After training, DNN-based solutions can provide results or predictions significantly more rapidly than numerical methods. The Runge–Kutta approach is based on the underlying mathematical model and its assumptions in contrast, once a DNN model is trained on a variety of significant and diverse data, it may be able to generalize to a wide range of problems.

**Fig. 6:**
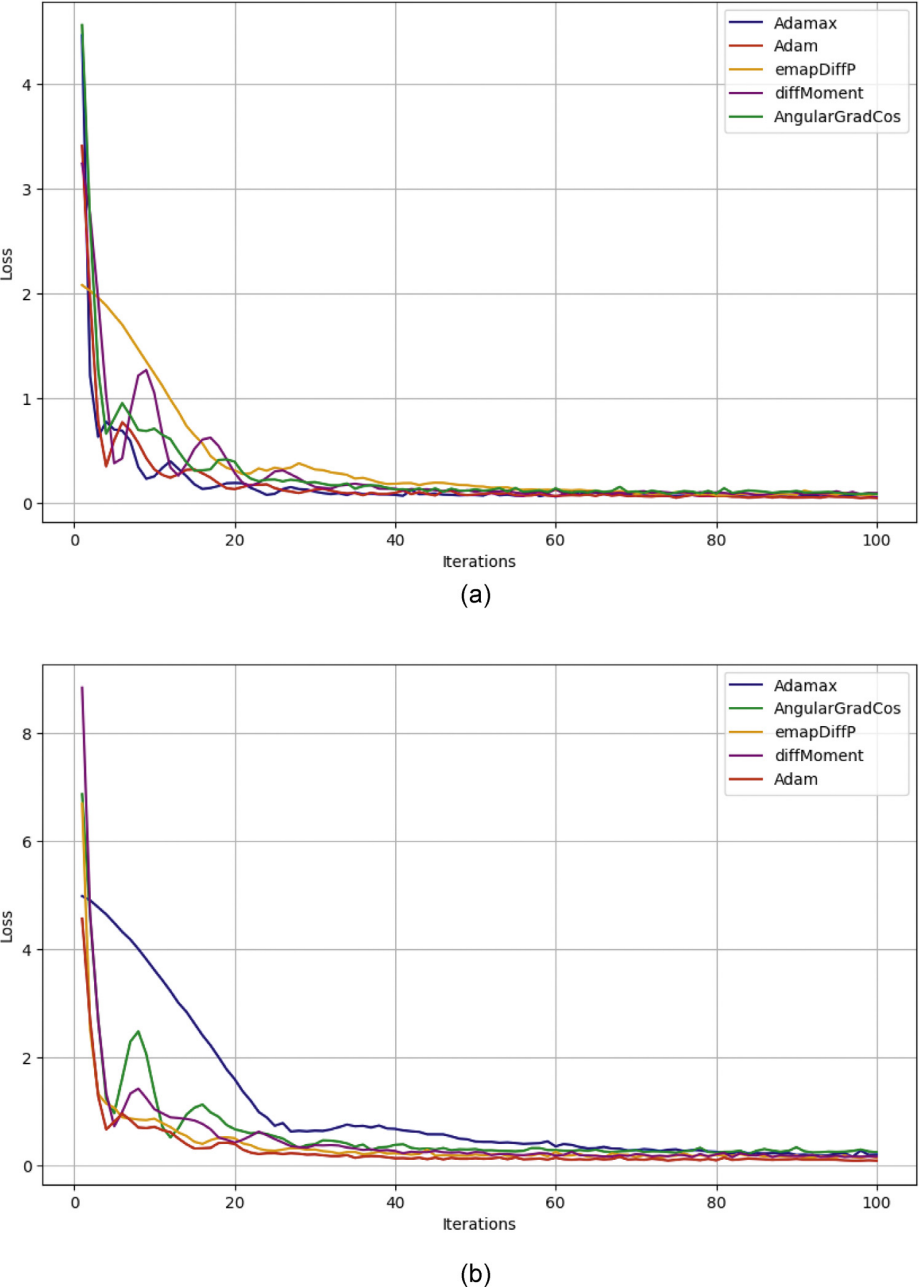
(a) Comparison of different optimizers in case of electrically actuated microbeam. (b) Comparison of different optimizers in case of multiwalled carbon nanotubes.

In this section, we apply different optimizers such as Adamax, Adam, emapDiffP, DiffMoment, AngularGradCos, to analyze the performance of these optimizers. The considered optimizers all have the same fundamental configurations. The learning rate is initially set as 0.001 and the decay rate of first and second moment is 0.9 and 0.999 respectively. The number of iterations is set to 200. In Fig. 6a, b is shown the result of loss values of different optimizers with respect to iterations. It is noticed that all the optimizers have able to gain zero loss values, but Adam achieved the zero loss values faster than the other and never overshot further. Moreover, the comparison of these optimizers in the form of table is also presented, which shows the suitability and effectiveness of suggested method (Table 1, Table 2).

**Table 1:** The loss values of different optimizers of MEMS in case of electrically actuated microbeam.

No. of iterations	emapDiff	DiffMoment	AngularGradCos	Adamax	Adam
100	9.04e-02	9.64e-02	1.03e-01	6.56e-02	4.51e-02
500	1.15e-03	6.47e-02	2.89e-04	2.20e-02	2.43e-04
1000	1.72e-04	1.86e-02	1.77e-04	5.87e-05	5.28e-05
2000	4.96e-05	3.44e-03	2.81e-04	1.66e-05	5.78e-05
4000	3.22e-05	1.84e-03	2.08e-04	9.19e-05	4.80e-05
6000	7.05e-04	1.70e-03	8.90e-03	1.60e-04	6.40e-05
8000	1.03e-04	1.50e-03	4.69e-05	4.87e-05	2.41e-05

**Table 2:** The loss values of different optimizers of MEMS in case of multiwalled carbon nanotubes.

No. of iterations	emapDiff	DiffMoment	AngularGradCos	Adamax	Adam
100	1.41e-01	1.50e-01	1.41e-01	2.04e-01	1.91e-01
500	5.30e-03	1.81e-03	3.35e-03	6.78e-02	2.08e-03
1000	1.82e-03	2.69e-03	2.59e-03	1.63e-02	3.78e-03
2000	1.47e-03	1.52e-03	1.89e-03	3.57e-03	1.38e-03
4000	2.75e-03	2.68e-03	2.86e-04	2.12e-03	6.05e-04
6000	5.87e-04	9.06e-04	1.97e-05	2.03e-03	9.25e-05
8000	1.27e-05	2.15e-04	1.21e-03	1.35e-03	4.56e-05

## Conclusions

Microelectromechanical systems (MEMS) have demonstrated a lot of interest due to their interesting properties, which include small size, excellent reliability, batch fabrication, and low energy consumption. The oscillatory behavior of these systems becomes very challenging because these systems have large nonlinearities. The suggested DNN approach has shown to be an effective tool for finding the approximating the solution of MEMS. We have shown through a series of investigations that even with changes to the parametric variables, the DNN design can accurately capture the nonlinear dynamics and bifurcation behavior of the system. In case of error computation, it is very useful to visualize how each parametric value affects the dependent variable. The graphical findings highlight the deviations and modifications that the model found. These outcomes demonstrate that the DNN method can be a useful tool for comprehending and evaluating the amplitude-frequency relationship in MEMS. The validity of our suggested model is confirmed by the outstanding agreement of the result obtained by DNN and the already existing numerical method RK4. Since the training and validation loss graphs are convergent, it is easy to conclude that the resulting model is robust. Finally, it is important to note that DNN is a reliable, and computationally faster method. As a result, this approach can also be used to solve different nonlinear MEMS problems involving mixed derivatives, for example the doubly clamped cylindrical nanowires based on Casimir attraction the double-sided nano-bridge that takes centrifugal force and rarefied gas flow. It can also be applied to nonlinear oscillators that have fractional or fractal derivatives.

## Limitations

Not Applicable

## Ethics authors statements

The platforms’ data redistribution policies were complied with.

## Funding statement

This research received no external funding

## Supplementary material and/or additional information

Not applicable.

## CRediT authorship contribution statement

**Muhammad Amir:** Conceptualization, Methodology, Writing – original draft. **Jamshaid Ul Rahman:** Conceptualization, Methodology, Writing – original draft. **Ali Hasan Ali:** Visualization, Investigation, Software. **Ali Raza:** Visualization, Resources, Validation, Writing – review & editing. **Zaid Ameen Abduljabbar:** Resources, Validation, Writing – review & editing. **Husam A. Neamah:** Resources, Validation, Writing – review & editing.

## Declaration of competing interest

The authors declare that they have no known competing financial interests or personal relationships that could have appeared to influence the work reported in this paper.

## Data Availability

Data will be made available on request.

## Appendix A

$g_{0}=\int_{0}^{1}\psi^{2}d\eta ,$

$g_{1}=-2\int_{0}^{1}\psi^{4}d\eta ,$

$g_{2}=\int_{0}^{1}\psi^{6}d\eta ,$

$g_{3}=\int_{0}^{1}\left(\psi \psi^{\prime \prime \prime }\prime -N\psi \psi \prime \prime -V^{2}\psi^{2}\right)d\eta ,\;$

$g_{4}=\int_{0}^{1}\left(\left(-2\psi^{3}\psi^{\prime \prime \prime }\prime +2N\psi^{3}\psi \prime \prime -\alpha \psi \psi \prime \prime \right)\int_{0}^{1}{\psi^{\prime }}^{2}d\eta \right)d\eta ,$

$g_{5}=\int_{0}^{1}\left(\left(\psi^{3}\psi^{\prime \prime \prime }\prime -N\psi^{5}-2\alpha \psi^{3}\psi \prime \prime \right)\int_{0}^{1}{\psi^{\prime }}^{2}d\eta \right)d\eta ,$

$g_{6}=-\int_{0}^{1}\left(\alpha \psi^{5}\psi \prime \prime \int_{0}^{1}{\psi \prime }^{2}d\eta \right)d\eta .$

## References

[1] Li X.X., He J.H. (2020). Bubble electrospinning with an auxiliary electrode and an auxiliary air flow. Recent. Pat. Nanotechnol..

[2] Yu D.N., Tian D., Zhou C.J., He J.H. (2019). Wetting and supercontraction properties of spider-based nanofibers. Thermal Sci..

[3] Li X., Qiang J., Wan Y., Wang H., Gao W. (2019). The effect of sonic vibration on electrospun fiber mats. J. Low Freq. Noise, Vibrat. Active Control.

[4] Li X.X., He J.H. (2019). Nanoscale adhesion and attachment oscillation under the geometric potential. Part 1: the formation mechanism of nanofiber membrane in the electrospinning. Results. Phys..

[5] Li X., Li Y., Li Y., He J. (2020). Gecko-like adhesion in the electrospinning process. Results. Phys..

[6] He C.H., He J.H., Sedighi H.M. (2020). Fangzhu (方诸): an ancient Chinese nanotechnology for water collection from air: history, mathematical insight, promises, and challenges. Math. Methods Appl. Sci..

[7] Liu H.Y., Li Z., Yao Y. (2019). A fractional nonlinear system for release oscillation of silver ions from hollow fibers. J. Low Freq. Noise, Vibrat. Active Control.

[8] Jin X., Liu M., Pan F., Li Y., Fan J. (2019). Low frequency of a deforming capillary vibration, part 1: mathematical model. J. Low Freq. Noise, Vibrat. Active Control.

[9] Malik S., Muhammad K., Waheed Y. (2023). Nanotechnology: a revolution in modern industry. Molecules..

[10] Torkashvand Z., Shayeganfar F., Ramazani A. (2024). Nanomaterials based micro/nanoelectromechanical system (MEMS and NEMS) devices. Micromachines. (Basel).

[11] Tovar-Lopez F.J. (2023). Recent progress in micro-and nanotechnology-enabled sensors for biomedical and environmental challenges. Sensors.

[12] Nathanson H.C., Newell W.E., Wickstrom R.A., Davis J.R. (1967). The resonant gate transistor. IEEe Trans. Electron. Devices.

[13] Taylor, G.I. (1968). The coalescence of closely spaced drops when they are at different electric potentials. *Proceedings of the Royal Society of London. Series A. Mathematical and Physical Sciences*, 306(1487), 423–434.

[14] Rebeiz G.M. (2004).

[15] Hung E.S., Senturia S.D. (1999). Extending the travel range of analog-tuned electrostatic actuators. J. Microelectromech. Syst..

[16] Qiu J., Lang J.H., Slocum A.H. (2004). A curved-beam bistable mechanism. J. Microelectromech. Syst..

[17] Michael A., Kwok C.Y. (2006). Design criteria for bi-stable behavior in a buckled multi-layered MEMS bridge. J. Micromech. Microeng..

[18] Ghiathinejad N., Zand M.M., Haghighi-Yazdi M., Dargazany R. (2019). Dynamic pull-in and snap-through behavior of electrostatically actuated micro-mechanical memories considering thermoelastic damping. Mech. Adv. Mater. Struct..

[19] Ghashami G., Zand M.M., Mahnama M., Allaei S.M.V., López-Suárez M. (2024). The effects of physical morphologies and strain rate on piezoelectric potential of boron nitride nanotubes: a molecular dynamics simulation. Nanotechnology..

[20] Sulfridge M., Saif T., Miller N., Meinhart M. (2004). Nonlinear dynamic study of a bistable MEMS: model and experiment. J. Microelectromech. Syst..

[21] Chen, X., & Meguid, S.A. (2015). Snap-through buckling of initially curved microbeam subject to an electrostatic force. *Proceedings of The Royal Society A: Mathematical, Physical and Engineering Sciences*, 471(2177), 20150072. 10.1098/rspa.2015.0072 PMC4985042 27547104

[22] Anjum N., He J.H., He C.H., Gepreel K.A. (2023). Variational iteration method for prediction of the pull-in instability condition of micro/nanoelectromechanical systems. Phys. Mesomech..

[23] Gorecki C., Bargiel S. (2020). MEMS scanning mirrors for optical coherence tomography. Photonics..

[24] Ghanbari M., Rezazadeh G. (2021). A MEMS-based methodology for measurement of effective density and viscosity of nanofluids. Europ. J. Mechan.-B/Fluids.

[25] Faedo N., Dores Piuma F.J., Giorgi G., Ringwood J.V. (2020). Nonlinear model reduction for wave energy systems: a moment-matching-based approach. Nonlinear. Dyn..

[26] Nuñez D., Perdomo O., Rivera A. (2019). On the stability of periodic solutions with defined sign in MEMS via lower and upper solutions. Nonlinear Anal..

[27] He J.H., Nurakhmetov D., Skrzypacz P., Wei D. (2021). Dynamic pull-in for micro–electromechanical device with a current-carrying conductor. J. Low Freq. Noise, Vibrat. Active Control.

[28] Amir M., Ashraf A., Haider J.A. (2024). The variational iteration method for a pendulum with a combined translational and rotational system. Acta Mechanica et Automatica.

[29] Qian Y.H., Pan J.L., Qiang Y., Wang J.S. (2019). The spreading residue harmonic balance method for studying the doubly clamped beam-type N/MEMS subjected to the van der Waals attraction. J. Low Freq. Noise, Vibrat. Active Control.

[30] He J.H. (2019). The simplest approach to nonlinear oscillators. Results. Phys..

[31] Javidi R., Rezaei B., Moghimi Zand M. (2023). Nonlinear dynamics of a beam subjected to a moving mass and resting on a viscoelastic foundation using optimal homotopy analysis method. Int. J. Struct. Stabil. Dyn..

[32] Kacem N., Baguet S., Hentz S., Dufour R. (2011). Computational and quasi-analytical models for non-linear vibrations of resonant MEMS and NEMS sensors. Int. J. Non. Linear. Mech..

[33] Vyasarayani, C.P., Abdel-Rahman, E.M., McPhee, J., & Birkett, S. (2011). Modeling MEMS resonators past pull-in.

[34] Abedinnasab M.H., Eigoli A.K., Zohoor H., Vossoughi G. (2011). On the influence of centerline strain on the stability of a bimorph piezo-actuated microbeam. Scientia Iranica.

[35] Yan Y., Wang W.Q., Zhang L.X. (2009). Dynamical behaviors of fluid-conveyed multi-walled carbon nanotubes. Appl. Math Model..

[36] Yun K., Choi J., Kim S.K., Song O. (2012). Flow-induced vibration and stability analysis of multi-wall carbon nanotubes. J. Mechan. Sci. Technol..

[37] Huang K., Qu B., Xu W., Yao J. (2022). Nonlocal Euler–Bernoulli beam theories with material nonlinearity and their application to single-walled carbon nanotubes. Nonlinear. Dyn..

[38] Tang W., Anjum N., He J.H. (2023). Variational iteration method for the nanobeams-based N/MEMS system. MethodsX..

[39] Vassilev V.M., Valchev G.S. (2024). Dynamics and stability of double-walled carbon nanotube cantilevers conveying fluid in an elastic medium. Dynamics..

[40] Lu L., Meng X., Mao Z., Karniadakis G.E. (2021). DeepXDE: a deep learning library for solving differential equations. SIAM Rev..

[41] Chen F., Sondak D., Protopapas P., Mattheakis M., Liu S., Agarwal D., Di Giovanni M. (2020). Neurodiffeq: a python package for solving differential equations with neural networks. J. Open. Source Softw..

[42] McFall K.S., Mahan J.R. (2009). Artificial neural network method for solution of boundary value problems with exact satisfaction of arbitrary boundary conditions. IEEe Trans. Neural Netw..

[43] Kumar M., Yadav N. (2011). Multilayer perceptrons and radial basis function neural network methods for the solution of differential equations: a survey. Comput. Mathem. Applic..

[44] Mall S., Chakraverty S. (2015). Numerical solution of nonlinear singular initial value problems of Emden–Fowler type using Chebyshev Neural Network method. Neurocomputing..

[45] Sahoo A.K., Chakraverty S. (2023). A neural network approach for the solution of Van der Pol-Mathieu-Duffing oscillator model. Evol. Intell..

[46] Ul Rahman J., Danish S., Lu D. (2023). Deep Neural network-based simulation of Sel’kov model in glycolysis: a comprehensive analysis. Mathematics.

[47] Ketkar N., Santana E. (2017).

[48] Krentel, M.W. (1986, November). The complexity of optimization problems. In *Proceedings of the eighteenth annual ACM symposium on Theory of computing* (pp. 69–76).

[49] Sharma S., Sharma S., Athaiya A. (2017). Activation functions in neural networks. Towards Data, Sci..

[50] Tian Y., Su D., Lauria S., Liu X. (2022). Recent advances on loss functions in deep learning for computer vision. Neurocomputing..

[51] Qi J., Du J., Siniscalchi S.M., Ma X., Lee C.H. (2020). On mean absolute error for deep neural network based vector-to-vector regression. IEEe Signal. Process. Lett..

[52] Wright L.G., Onodera T., Stein M.M., Wang T., Schachter D.T., Hu Z., McMahon P.L. (2022). Deep physical neural networks trained with backpropagation. Nature.

[53] Yong, H., Huang, J., Hua, X., & Zhang, L. (2020). Gradient centralization: a new optimization technique for deep neural networks. In *Computer Vision–ECCV 2020: 16th European Conference, Glasgow, UK, August 23–28, 2020, Proceedings, Part I 16* (pp. 635–652). Springer International Publishing.

[54] El Helou M., Süsstrunk S. (2020). Blind universal bayesian image denoising with gaussian noise level learning. IEEE Transac. Image Process..

[55] Sedighi H.M., Daneshmand F. (2014). Static and dynamic pull-in instability of multi-walled carbon nanotube probes by He’s iteration perturbation method. J. Mechan. Sci. Technol..

